# Killing capacity analysis of tumor-infiltrating cytotoxic lymphocytes and impact on lymph node metastasis in differentiated papillary carcinoma of thyroid with the BRAF V600E mutation

**DOI:** 10.1186/s13000-024-01454-9

**Published:** 2024-02-10

**Authors:** Xiaogang Liu, Honggang Liu, Lu Wang, Yubing Han, Linghong Kong, Xinpeng Zhang

**Affiliations:** 1 Department of Pathology, Beijing Tongren Hospital, Beijing Key Laboratory of Head and Neck Molecular Diagnostic Pathology, Capital Medical University, Beijing, 100730 China; 2 Department of Pathology, Beijing Chuiyangliu Hospital, Beijing, 100022 China

**Keywords:** Papillary carcinoma of the thyroid, Cytotoxic lymphocytes, Perforin, Granzyme B, Lymph node metastasis

## Abstract

**Background:**

Cytotoxic lymphocytes (CLs) express potent toxins, including perforin (P) and granzyme-B (G), which brings about target cell death. The purpose of this study was to evaluate the killing capacity of tumor-infiltrating CLs by means of P and G analysis, and explore the association with lymph node metastasis in papillary carcinoma of thyroid (PTC) without Hashimoto’s thyroiditis (HT).

**Methods:**

Infiltration of lymphocytes in PTC was observed in frozen sections. Both fresh tumor tissues and paracancerous tissues with lymphocyte infiltration were collected and prepared into a single cell suspension. Flow cytometry was used to detect the percentages of CD3^+^P^+^, CD3^+^G^+^, CD8^+^P^+^, and CD8^+^G^+^ T lymphocytes (TLs) and CD16-CD56^+^P^+^ and CD16-CD56^+^G^+^ natural killer (NK) cells. Finally, we investigated differential expression of P and G in NK cells and cytotoxic T lymphocytes (CTLs) in paired tumor tissues (group T, *n* = 44) and paracancerous tissues (group N, *n* = 44) from patients with PTC with the BRAF V600E mutation. Furthermore, patients were divided into two groups according to whether cervical central lymph node metastasis (CCLNM) existed: group A (with lymph node metastases, *n* = 27) and group B (with nonlymph node metastases, *n* = 17). Patients were also divided into three groups according to the total number of positive CCLNM: group B, group C (with low-level lymph node metastases, less than 5, *n* = 17) and group D (with high-level lymph node metastases, no less than 5, *n* = 10).

**Results:**

The percentage of CD3^+^P^+^ CTLs was significantly higher in group N than in group T (*P* < 0.05). The percentage of CD8^+^G^+^ CTLs was significantly higher in group T than in group N (*P* < 0.05). The percentages of CD3^+^G^+^, CD16-CD56^+^P^+^and CD16-CD56^+^G^+^ NK cells showed no significant difference in either group T or group N (*P* > 0.05). The percentages of CD3^+^P^+^ CTLs in group A and group C were significantly higher in the paracancerous tissue than in the tumor tissue (*P* < 0.05). The percentages of CD8^+^G^+^ CTLs in group A and group C were significantly higher in the tumor tissues than in the paracancerous tissues (*P* < 0.05). The percentage of CD16-CD56^+^G^+^ NK cells in group D was significantly higher in the tumor tissues than in the paracancerous tissues (*P* < 0.05).

**Conclusions:**

The killing capacity of infiltrating CLs in PTC differed between tumor tissues and paracancerous tissues. In cases with CCLNM, higher expression of CD16-CD56^+^G^+^ NK cells in tumor tissues may be associated with a high risk of lymph node metastasis.

## Introduce

Tumors derived from the thyroid gland are the most common endocrine tumors [1], and the incidence of PTC has been rising throughout the world [2, 3]. Current studies suggest that immune cells may play an important role in tumor behavior, including tumor initiation, progression, metastasis and immunotherapy effects [4, 5]. Studies about the immune response in PTC have also been increasingly carried out, which may lead to novel immunotherapeutic approaches in thyroid cancer [6–8]. One study showed that expression of PD-1 in T lymphocytes in peripheral blood significantly differed in PTC, nodular goiter (NG) and HP patients. T-cell exhaustion is considered a risk factor for lymph node metastasis [9]. Research has shown that many tumor-infiltrating FoxP3^+^ Treg cells are found in primary PTC and metastatic lymph node tissues. A higher percentage of Treg cells in both peripheral blood and tumor tissues may be related to tumor aggressiveness [10]. In addition, more CD3^+^CD4^−^CD8^−^ double-negative (DN) T lymphocytes infiltrate thyroid cancer tissues more than benign nodules, such as in NG, HT, and Graves’ disease. The cutoff value of > 9.14% DN T lymphocytes has been proposed for early diagnosis of thyroid cancer with 100% specificity [11]. The above results indicate new diagnostic and immunotherapeutic targets for PTC.

The purpose of this study was to research the killing capacity of tumor-infiltrating NK cells and CTLs in the tumor microenvironment (TME) and evaluate the association between CLs and CCLNM in PTC, which may provide a novel immunotherapeutic strategy for PTC patients.

## Materials and methods

### Patients

The present study was approved by the Beijing Chuiyangliu Hospital ethical committee (Version number: SOP-CYLIRB-2.0). The criteria for inclusion in this study included solitary classic PTC nodules ranging from 1 to 1.8 cm in greatest dimension limited to the thyroid (T1b), a number of cervical central lymph nodes resected of no less than 5, and the BRAF V600E mutation positivity. The exclusion criteria were lack of lymphocyte infiltration in tumor tissues or paracancerous tissues and those with Hashimoto’s thyroid or other malignant tumors.

### Tissue preparation

**Fig. 1:**
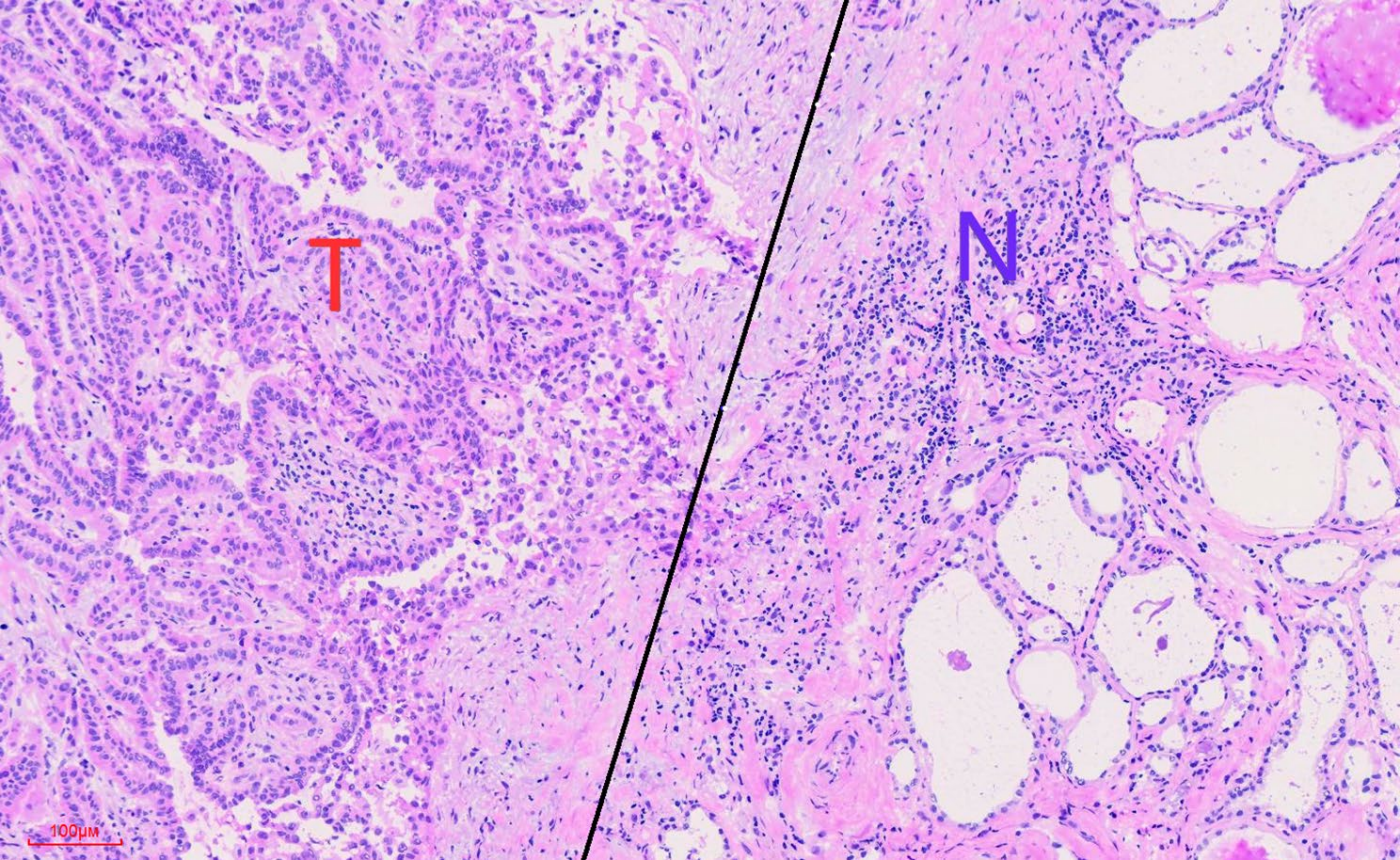
Lymphocytes infiltrated in both tumor tissues (T) and paracancerous tissues (N) by frozen sectioning

The remaining fresh tissue samples after pathology sampling of classic PTC diagnosed by frozen sectioning were collected in the Department of Pathology of Beijing Chuiyangliu Hospital. Tumor tissues and paracancerous tissues with lymphocyte infiltration confirmed by frozen sections [Fig. 1] were collected separately and immersed in a Petri dish containing phosphate-buffered saline (PBS). Then, the tissue was sheared with phthalmic scissors, transferred with PBS to a 5 mL tissue homogenizer and minced into a small tissue homogenate. The tissue homogenate was filtered through a 300 mesh stainless steel strainer. The cell suspension was collected in a 15 mL centrifuge tube with PBS and centrifuged at 300 × g for 5 min. After the supernatant was removed, the cells were resuspended in 300 µL of PBS, and flow cytometry analysis was performed.

### Flow cytometry analysis

**Fig. 2:**
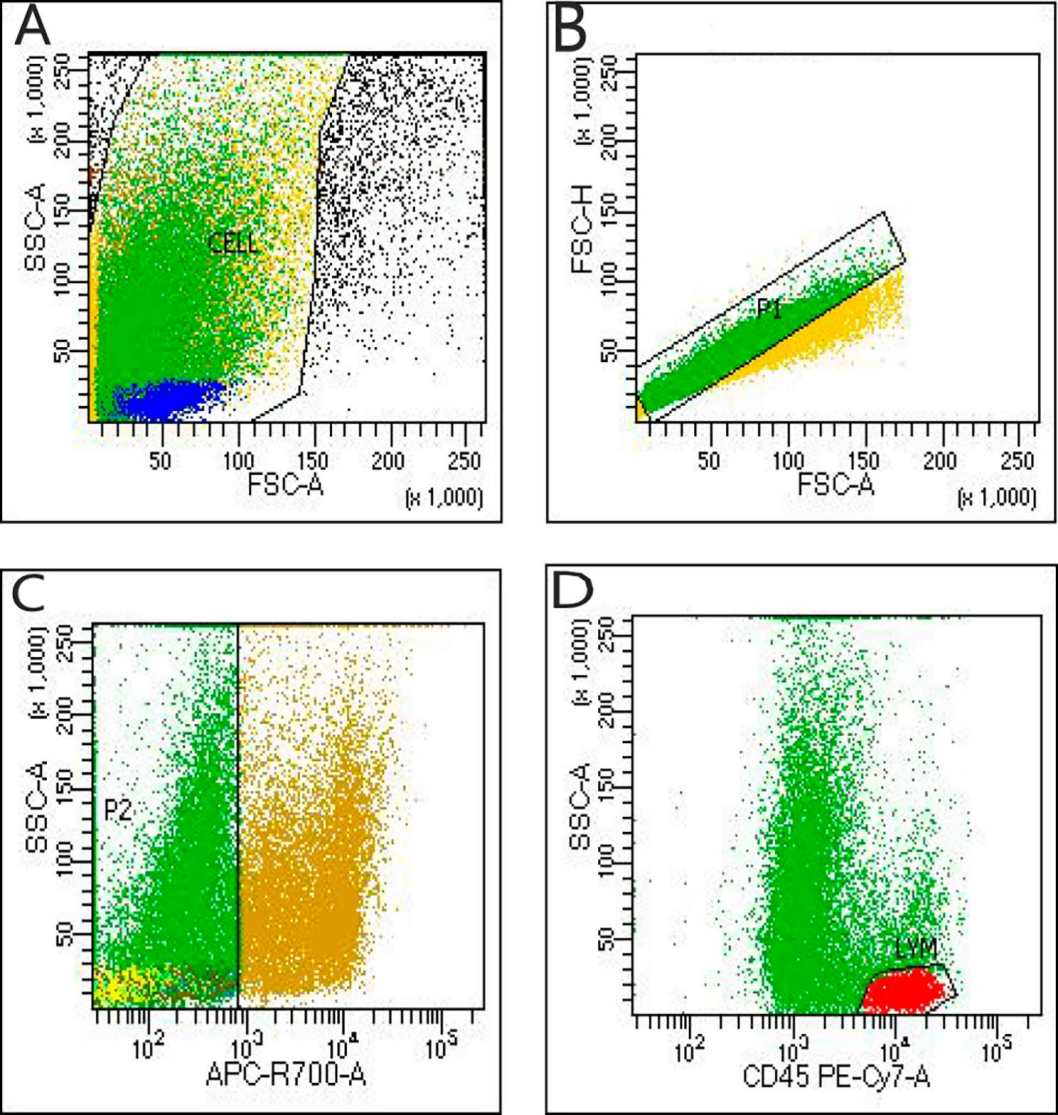
(**A**) Select cell gating by FSC/SSC was performed. (**B**) P1 was plotted by FSC-A/FSC-H. (**C**) P2 was plotted by APCR700/SSC. (**D**) The CD45/SSA gating strategy was used for the next analysis

**Fig. 3:**
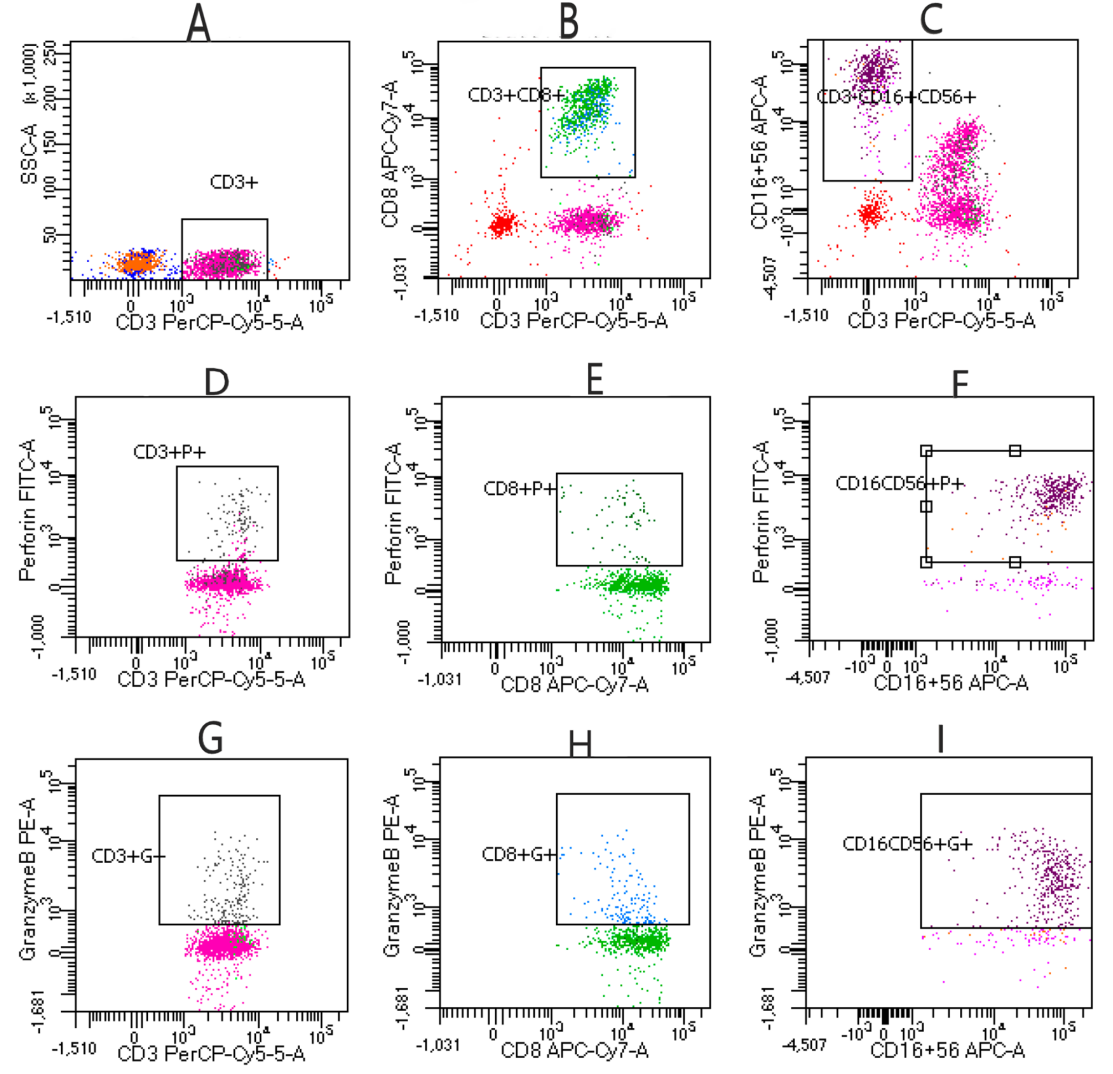
(**A**) CD3^+^ T lymphocytes; (**B**) CD8^+^ T lymphocytes; (**C**) CD3^−^CD16-CD56^+^ NK cells; (**D**) CD3^+^Perforin^+^ T lymphocytes; (**E**) CD8^+^perforin^+^ T lymphocytes; (**F**) CD16-CD56^+^perforin^+^ NK cells; (**G**) CD3^+^granzyme-B^+^ T lymphocytes; (**H**) CD8^+^granzyme-B^+^ T lymphocytes; (**I**) CD16CD56^+^granzyme-B^+^ NK cells

Antibodies used for flow cytometry included CD45 (PE-Cy7), CD3 (PerCP), CD8 (APC-Cy7), CD16 (APC), CD56 (APC), perforin and granzyme B, all from RAISE CARE Biotechnology Co. Ltd. (Qingdao, China). Data were analyzed using a BD FACSCanto analyzer. A 50 µL cell suspension and 25 µL premix antibody reagent including CD45, CD3, CD8, CD16, and CD56 were incubated in the dark for 15 min at room temperature. Then, 120 µL fixative and 2 mL membrane break regent were added, and the sample was incubated in the dark for 15 min. The solution was centrifuged at 350 x g for 5 min, and the supernatant was removed. Then, 5 µL of granzyme B and perforin were added and incubated in the dark for 15 min. The samples were suspended in 200 µL of PBS and analyzed with a BD FACSCanto cytometer and BD FASCDiva 8.0.3 software. Selective cell gating by FSC/SSC was performed. In addition, P1 was plotted by using FSC-A/FSC-H to avoid adhesive, and P2 was further plotted by APCR700/SSC to avoid nonspecific dead cells. We plotted lymphocyte subsets with high CD45 and low SSC phenotype characteristics [Fig. 2], and at least 3000 gated events were measured in every sample. We plotted CD3/SSC and selected CD3^+^ cells. By following the percentages of CD3^+^P^+^, CD3^+^G^+^, CD8^+^P^+^, and CD8^+^G^+^ T-cell populations, the percentages of CD3^−^CD16-CD56^+^P^+^ and CD3^−^CD16-CD56^+^G^+^ NK cell populations were obtained [Fig. 3].

### Molecular analysis

DNA was extracted from the same surgical paraffin-embedded tissue blocks using DNA Sample Preparation Kit (Roche USA) following the manufacturer’s instructions. Exon 15 mutation of the BRAF gene (V600E) was assessed using a Cobus 4800 BRAF V600 Mutation Test Kit (Roche, USA) and a Cobas z480 following the manufacturer’s instructions at the Molecular Pathology Laboratory of Chuiyangliu Hospital.

### Statistical analysis

Statistical analysis was performed using GraphPad Prism version 9.5.1 software. The paired sample t test, independent t test and one-way analysis of variance (ANOVA) were used for statistical comparisons. The significance level was set at *P* < 0.05.

## Results

### Patient population characteristics

Based on the above criteria, 44 PTC patients (35 female) were included from May to September 2023. The median age was 43.43 (range 23–71) years. All cases were divided into two paired groups: the tumor group (group T, *n* = 44) and the paracancerous group (group N, *n* = 44). Patients were divided into two groups according to whether CCLNM existed: group A (with lymph node metastases, *n* = 27) and group B (with nonlymph node metastases, *n* = 17). Furthermore, patients were divided into three groups according to the total number of positive CCLNM: group B, group C (with low-level lymph node metastases, less than 5, *n* = 17) and group D (with high-level lymph node metastases, no less than 5, *n* = 10).

### Flow cytometry results

#### The killing capacity characteristics of NK cell and CTL subpopulations in the core tumor tissues and paracancerous tissues of PTC

**Fig. 4:**
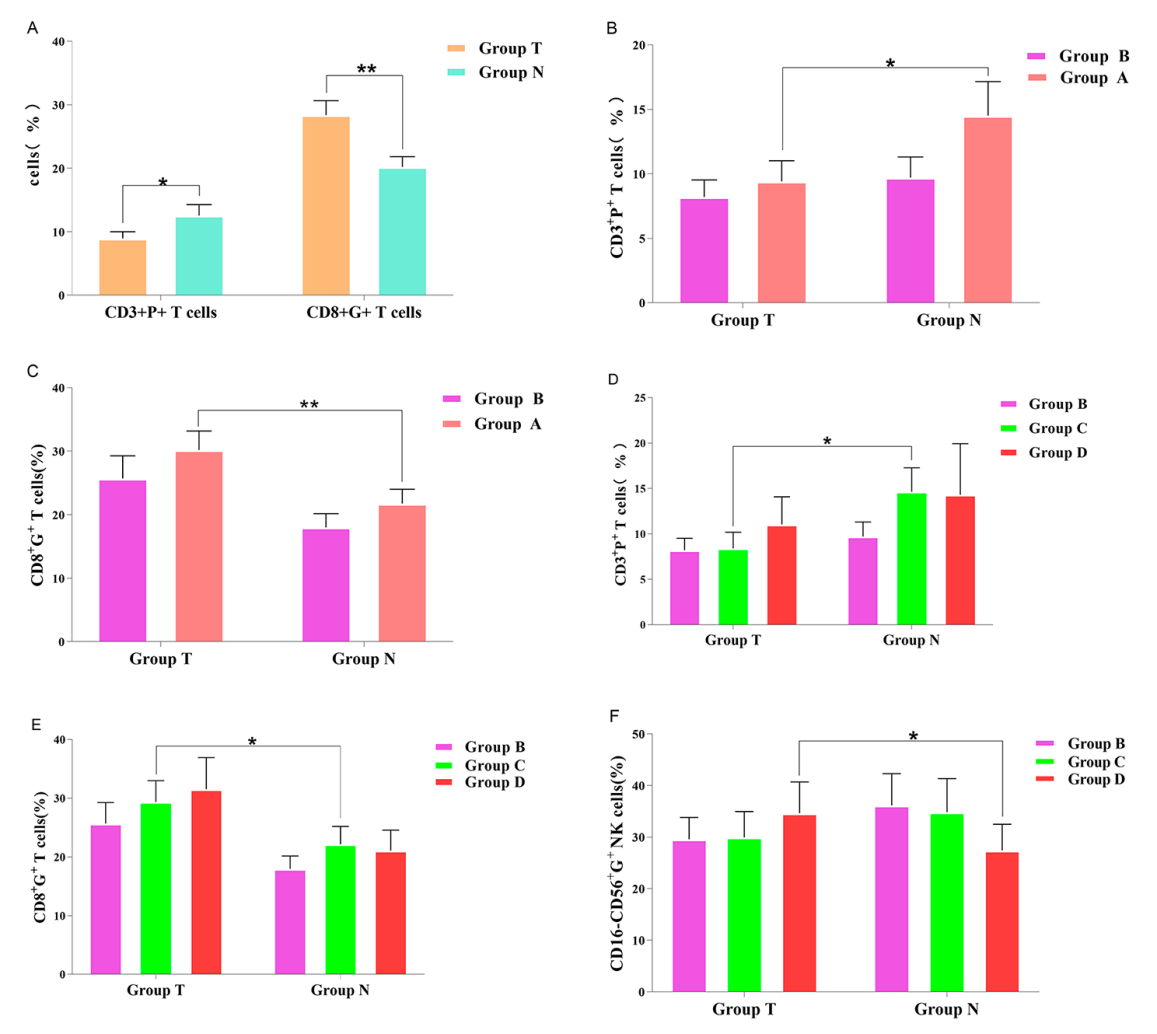
(**A**) The percentage of the CD3^+^P^+^ T-cell subset in group T was significantly lower than that in group N, and the percentage of the CD8^+^G^+^ T-cell subset in group T was significantly higher than that in group N (*P* < 0.05). (**B**) The percentage of the CD3^+^P^+^ T-cell subset in group A in tumor tissues was significantly lower than that in paracancerous tissues (*P* < 0.05). (**C**) The percentage of the CD8^+^G^+^ T-cell subset in group A in tumor tissues was significantly higher than that in paracancerous tissues (*P* < 0.05). (**D**) The percentage of the CD3^+^P^+^ T-cell subset in group C in tumor tissues was significantly lower than that in paracancerous tissues (*P* < 0.05). (**E**) The percentage of the CD8^+^G^+^ T-cell subpopulation in group C in tumor tissue was significantly higher than that in paracancerous tissues (*P* < 0.05). (**F**) The percentage of CD16-CD56^+^G^+^ NK cells subset of the group C in the tumor tissue was significantly higher than that in the paracancerous tissue (*P* < 0.05)

We examined expression of CD3^+^P^+^, CD3^+^G^+^, CD8^+^P^+^, and CD8^+^G^+^ CTL subpopulations and CD16-CD56^+^P^+^ and CD16-CD56^+^G^+^ NK cell subpopulations in group T and group N PTC (Table 1). The results showed that the percentage of CD3^+^P^+^ CTL subpopulations in group T was significantly lower than that in group N (*P* < 0.05) but that the percentage of CD8^+^G^+^ CTL subpopulations in group T was significantly higher than that in group N (*P* < 0.05, Fig. 4A) according to a paired sample t test. In addition, expression of CD3^+^G^+^ and CD8^+^P^+^ CTL subpopulations and CD16-CD56^+^P^+^ and CD16-CD56^+^G^+^ NK cell subpopulations between the two groups was not significantly different (*P* > 0.05).

#### Relative differential killing capacity of NK cell and CTL subpopulations in core tumor tissues and paracancerous tissues with or without CCLNM in PTC

We examined expression of CD3^+^P^+^, CD3^+^G^+^, CD8^+^P^+^, and CD8^+^G^+^ CTL subpopulations and CD16-CD56^+^P^+^ and CD16-CD56^+^G^+^ NK cell subpopulations in groups A and B of PTC (Table 2). The results showed that the percentage of CD3^+^P^+^ CTL subpopulations in group A in the tumor tissues was significantly lower than that in the paracancerous tissues (*P* < 0.05, Fig. 4B) but that the percentage of CD8^+^G^+^ CTL subpopulations in group A in the tumor tissues was significantly higher than that in the paracancerous tissues (*P* < 0.05, Fig. 4C) by a paired sample t test. In addition, the percentages of CD3^+^G^+^ and CD8^+^P^+^ CTLs and CD16-CD56^+^P^+^ and CD16-CD56^+^G^+^ NK cells in group A were not significantly different between the tumor tissues and paracancerous tissues (*P* > 0.05). No significant differences in any data in group B were observed between the tumor tissues and paracancerous tissues (*P* > 0.05). There were no significant differences between group A and group B of all indexes in the tumor tissues or paracancerous tissues (*P* > 0.05) according to the independent samples t test.

#### Relative differential killing capacity of NK cell and CTL subpopulations in core tumor tissues and paracancerous tissues with varying levels of CCLNM in PTC

We examined expression of CD3^+^P^+^, CD3^+^G^+^, CD8^+^P^+^, and CD8^+^G^+^ CTL subpopulations and CD16-CD56^+^P^+^ and CD16-CD56^+^G^+^ NK cell subpopulations in groups B, C and D of PTC (Table 3). The results showed that the percentage of CD3^+^P^+^ CTL subpopulations in group C in the tumor tissues was significantly lower than that in the paracancerous tissues (*P* < 0.05, Fig. 4E) but that the percentage of CD8^+^G^+^ CTL subpopulations in group C in the tumor tissues was significantly higher than that in the paracancerous tissues (*P* < 0.05, Fig. 4E) by a paired sample t test. In addition, the subpolulation of CD16-CD56^+^G^+^ NK cells in group C in the tumor tissues was significantly higher than that in the paracancerous tissues (*P* < 0.05, Fig. 3F). The results of ANOVA indicated that the percentage of NK cells and CTL subpopulations did not differ significantly in the tumor tissues or the paracancerous tissues among group B, group C and group D.

### Molecular analysis

In this study, BRAF V600E mutation positivity in PTC with lymphocyte infiltration characteristics was 83.9% (52/62). Forty-four PTC-positive patients were included in the final analysis.

**Table 1:** Killing capacity indexes of NK cells and CTLs in tumor tissues and paracancerous tissues

	Group T(*n* = 44)	Group N(*n* = 44)	t	P
CD3^+^P^+^(%)	8.93 ± 1.07	12.55 ± 1.71	-2.466	0.018*
CD3^+^G^+^(%)	20.8 ± 2.03	20.78 ± 2.71	0.01	0.992
CD8^+^P^+^(%)	8.75 ± 1.01	9.13 ± 1.07	-0.293	0.771
CD8^+^G^+^(%)	28.36 ± 2.28	20.21 ± 1.61	3.719	0.001*
CD16-CD56^+^P^+^(%)	29.35 ± 2.94	35.13 ± 3.66	-1.839	0.073
CD16-CD56^+^G^+^(%)	30.87 ± 2.81	33.69 ± 3.6	-1.159	0.253

Values are expressed as the mean ± standard error of the mean (SEM). Comparisons were made

using a paired t test. *Represents significant differences (*P* < 0.05)

**Table 2:** Relativity indexes of the differential killing capacity of NK cells and CTLs in tumor tissues and paracancerous tissues with or without CCLNM in PTC

	Group B(*n* = 17)	Group A(*n* = 27)	t	P
CD3^+^P^+^(%)_T	8.21 ± 1.32	9.42 ± 1.59	-0.552	0.584
CD3^+^P^+^(%)_N	9.71 ± 1.6	14.52 ± 2.64	-1.56	0.127
t	-1.068	-2.245		
p	0.3	0.034*		
CD3^+^G^+^(%)_T	19.66 ± 3.26	21.59 ± 2.63	-0.465	0.644
CD3^+^G^+^(%)_N	15.76 ± 2.75	24.25 ± 4.09	-1.565	0.125
t	1.842	-0.799		
p	0.083	0.432		
CD8^+^P^+^(%)_T	7.87 ± 1.43	9.37 ± 1.4	-0.725	0.472
CD8^+^P^+^(%)_N	9.56 ± 1.62	8.84 ± 1.43	0.326	0.746
t	-0.877	0.297		
p	0.393	0.769		
CD8^+^G^+^(%)_T	25.71 ± 3.56	30.2 ± 2.98	-0.969	0.338
CD8^+^G^+^(%)_N	18 ± 2.16	21.75 ± 2.26	-1.147	0.258
t	1.963	3.264		
p	0.066	0.003*		
CD16-CD56 + P+(%)_T	29.56 ± 4.38	29.2 ± 4.01	0.058	0.954
CD16-CD56 + P+(%)_N	37.49 ± 6.13	33.49 ± 4.59	0.532	0.598
t	-1.761	-0.985		
p	0.096	0.334		
CD16-CD56 + G+(%)_T	29.58 ± 4.23	31.76 ± 3.82	-0.378	0.708
CD16-CD56 + G+(%)_N	36.16 ± 6.13	31.98 ± 4.44	0.565	0.575
t	-1.895	-0.067		
p	0.075	0.947		

Values are expressed as the mean ± standard error of the mean (SEM). Within-group comparisons

were made using paired t tests, and comparisons between groups were made using independent

samples t test, *represents significant differences (*P* < 0.05)

**Table 3:** Relativity indexes of the differential killing capacity of NK cells and CTLs in tumor tissues and paracancerous tissues with varying levels of CCLNM in PTC

	Group B(*n* = 17)	Group C(*n* = 17)	Group D(*n* = 10)	F	P
CD3^+^P^+^(%)_T	8.21 ± 1.32	8.41 ± 1.78	11.05 ± 3.04	0.567	0.572
CD3^+^P^+^(%)_N	9.71 ± 1.6	14.64 ± 2.64	14.32 ± 5.62	0.954	0.394
t	-1.068	-2.249	-0.815		
p	0.3	0.04*	0.436		
CD3^+^G^+^(%)_T	19.66 ± 3.26	20.24 ± 3.37	23.76 ± 4.35	0.31	0.735
CD3^+^G^+^(%)_N	15.76 ± 2.75	20.7 ± 3.2	29.94 ± 9.36	2.097	0.136
t	1.842	-0.172	-0.807		
p	0.083	0.866	0.441		
CD8^+^P^+^(%)_T	7.87 ± 1.43	9.69 ± 1.91	8.85 ± 2.1	0.304	0.74
CD8^+^P^+^(%)_N	9.56 ± 1.62	9.82 ± 1.74	7.28 ± 2.53	0.439	0.648
t	-0.877	-0.059	0.519		
p	0.393	0.954	0.616		
CD8^+^G^+^(%)_T	25.71 ± 3.56	29.37 ± 3.62	31.54 ± 5.38	0.521	0.598
CD8^+^G^+^(%)_N	18 ± 2.16	22.16 ± 3.05	21.08 ± 3.48	0.675	0.515
t	1.963	2.869	1.88		
p	0.066	0.012*	0.093		
CD16-CD56^+^P^+^(%)_T	29.56 ± 4.38	25.96 ± 5.57	34.39 ± 5.34	0.565	0.573
CD16-CD56^+^P^+^(%)_N	37.49 ± 6.13	37.18 ± 6.65	27.59 ± 5.3	0.612	0.547
t	-1.761	-1.885	1.508		
p	0.096	0.079	0.166		
CD16-CD56^+^G^+^(%)_T	29.58 ± 4.23	29.96 ± 5.01	34.64 ± 6.07	0.257	0.775
CD16-CD56^+^G^+^(%)_N	36.16 ± 6.13	34.82 ± 6.52	27.45 ± 5.03	0.444	0.645
t	-1.895	-1.014	2.559		
p	0.075	0.327	0.031*		

Values are expressed as the mean ± standard error of the mean (SEM). Within-group comparisons

were made using paired t tests, and comparisons among groups were made using one-way

ANOVA, *represents significant differences (*P* < 0.05)

## Discussion

The tumor microenvironment (TME) comprises all noncancerous host cells in the tumor and its noncellular components, including molecular products [12–14]. Among them, cytotoxic lymphocytes are being investigated in the immune surveillance of cancer and potential immunotherapies, and the role of cytotoxic lymphocytes in defending against cancer has been appreciated [15, 16]. Cytotoxic lymphocytes include natural killer cells and cytotoxic T lymphocytes. NK cells, as innate immunity effector cells, play a significant role in inhibiting tumor progression [17]. Gogali F et al. found that the CD3^−^CD16^+^CD56^dim^ cell subpopulation had significant predominance compared to CD3^−^CD16^−^CD56^bright^ NK cells in blood samples among PTC, NG and healthy donors. The research showed that the CD3^−^CD16^−^CD56^bright^ NK cell subpopulation infiltrating tissue may be associated with PTC progression. CD16^−^CD56^bright^ NK cells with immunoregulatory functions were increased in the tumor microenvironment of PTC [18]. CD8^+^ T cells are a subpopulation of lymphocytes with the potential to kill tumor cells presenting major histocompatibility complex (MHC) class I molecules [19]. Both NK cells and cytotoxic T cells can secrete perforin and granzyme, which play key roles in innate and adaptive immune defense against cancer development [20, 21]. However, studies have found that tumors have specific mechanisms to induce tumor immunological tolerance by interfering with immune cell responses [22]. NK cell and CD8^+^ T-cell dysfunction and exhaustion have been confirmed in various cancers due to immunosuppression within the TME [23, 24], including thyroid cancer [25–27]. In this study, we investigated the killing capability of NK cells and CTLs to produce perforin and granzyme-B in different regions, including the core tumor infiltration region of the TME and the marginal infiltration zone of paracancerous tissues. Expression of CD3^+^P^+^, CD3^+^G^+^, CD8^+^P^+^, and CD8^+^G^+^ CTL subsets and CD16-CD56^+^P^+^ and CD16-CD56^+^G^+^ NK cell subsets was detected by flow cytometry. Our results showed that the percentages of granzyme-B and perforin were different in the TME and paracancerous tissues. The percentage of CD3^+^P^+^ T-cell subsets in the core tumor tissue was significantly lower than that in the paracancerous tissues, but the percentage of CD8^+^G^+^ T-cell subsets in the tumor tissues was significantly higher than that in the paracancerous tissues. Our study demonstrated that infiltrating cytotoxic lymphocytes in different regions of the TME and paracancerous tissues have different killing capacities.

Although PTC has favorable prognosis, CCLNM is common [28]. Indeed, CCLNM is known as a predictive risk factor for lateral lymph node metastasis and poor progression [29–31]. In this study, the CCLNM rate was 61.36%, consistent with a previous report [32].

The cutoff value of the positive number of CCLNMs ranged from 2 to 5 [33–36]. In this study, a positive number of CCLNM > 5 was accepted as a cutoff value for high risk. Patients with CCLNM (group A) were divided into group C (with low-level CCLNM) and group D (with high-level CCLNM). The percentage of the CD3^+^P^+^ T-cell subset in group C in the tumor tissues was significantly lower than that in the paracancerous tissues by a paired t test, though the percentage of the CD8^+^G^+^ T-cell subset in group C in the tumor tissues was significantly higher than that in the paracancerous tissues. In addition, the percentage of the CD16-CD56^+^G^+^ NK cell subset in group C in the tumor tissues was significantly higher than that in the paracancerous tissues. Higher expression of CD16-CD56^+^G^+^ NK cells in the core tumor region of PTC may be associated with a higher risk of lymph node metastasis, which may provide targeted immunotherapy prospects.

Hashimoto’s thyroiditis (HT), also known as chronic lymphocytic thyroiditis, is a common autoimmune disorder of the thyroid gland with lymphoplasmacytic cell infiltration into the stroma [37]. Current studies on the relationship between HT and PTC are controversial regarding whether HT affects PTC tumorigenesis and progression [38–40]. We found that distinguishing tumor-infiltrating immunocytes from innate immunocytes in PTC patients with HT differed. As a result, PTC patients with HT were excluded from this study.

The BRAF V600E mutation is known to be associated with aggressive tumor behavior, recurrence, and poor progression of PTC [41, 42]. Given the significant influence on PTC, in this study, cases with wild-type BRAF were excluded.

In summary, our study demonstrates that infiltration of cytotoxic lymphocytes in different regions of the TME and paracancerous tissues has different killing capacities and is associated with CCLNM. Further studies about the PD1/PD-L1 pathway, immune regulatory mechanism and PTC related to HT are needed, which may contribute to providing immunotherapeutic strategies for predicting progression.

## Data Availability

The datasets used during the current study are available from the corresponding author upon reasonable request.
